# A Pattern-Based Method for Medical Entity Recognition From Chinese Diagnostic Imaging Text

**DOI:** 10.3389/frai.2019.00001

**Published:** 2019-05-14

**Authors:** Zihong Liang, Junjie Chen, Zhaopeng Xu, Yuyang Chen, Tianyong Hao

**Affiliations:** ^1^School of Computer Science, South China Normal University, Guangzhou, China; ^2^Software College, Northeastern University, Shenyang, China

**Keywords:** medical named entity recognition, pattern-based strategy, information extraction, clinical text, natural language processing

## Abstract

**Background:** The identification of medical entities and relations from electronic medical records is a fundamental research issue for medical informatics. However, the task of extracting valuable knowledge from these records is challenging due to its high complexity. The accurate identification of entity and relation is still an open research problem in medical information extraction.

**Methods:** A pattern-based method for extracting certain tumor-related entities and attributes from Chinese unstructured diagnostic imaging text is proposed. This method is a composition of three steps. Firstly, an algorithm based on keyword matching is designed to obtain the primary sites of tumors. Then a set of regular expressions is applied to identify primary tumor size information. Finally, a set of rules is defined to acquire metastatic sites of tumors.

**Results:** Our method achieves a recall of 0.697, a precision of 0.825 and an F1 score of 0.755 using an overall weighted metric. For each of the extraction tasks, the F1 scores are 0.784, 0.822 and 0.740.

**Conclusions:** The method proves to be stable and robust with different amounts of testing data. It achieves a comparatively high performance in the CHIP 2018 open challenge, demonstrating its effectiveness in extracting tumor-related entities from Chinese diagnostic imaging text.

## 1. Introduction

Biomedical named entity recognition (NER) is a critical task for extracting patient information from medical diagnosis to support medical research and treatment decision making. The aim of NER is to locate and classify medical named entity mentions in unstructured text, such as treatment, symptom and so on (Demner-Fushman et al., 2009). Through NER, certain hidden information in the diagnosis could be dug out and further contribute to improving existing medical systems. More importantly, medical information processing systems that rely solely on structured data are unable to directly access such kinds of hidden information in the medical text. Building a practical NER system is not an easy task because of the complexity of medical text. In addition to the difficulty of accurately extracting named entities from unstructured medical text, another primary difficulty is the precise normalization of extracted named entities by mapping them to concepts (Buzhou Tang, 2018b).

There is an open challenge at the China Health Information Processing Conference (CHIP) 2018 aiming at competition regarding the methodology on clinical named entity recognition (Buzhou Tang, 2018a). It offers several subtasks addressing the information extraction problem focusing on the organ and size of cancer (Buzhou Tang, 2018b). The first subtask focuses on extracting organ entities of primary cancer and metastasis cancer. In the subtasks, models are expected to identify and normalize organ entities where primary cancer and metastasis caner exist. The second subtask is to identify the size of primary cancer mentioned in diagnosis. The challenge provides a training and a testing dataset. In the training data set, there are 600 entries of diagnoses, primary tumor site, primary tumor size and metastatic tumor site, mostly about lung cancer and breast cancer. The testing data containing 200 entries covers broader content compared to the training dataset.

Targeting the NER challenge on the standard CHIP 2018 dataset, we propose a pattern-based method which exploits background knowledge, discourse knowledge and domain-specific knowledge to extract the required entities. Based on the testing data, our method achieves F1 scores of 0.78, 0.82, and 0.74 on the primary tumor site, primary tumor size and metastatic tumor site identification tasks, respectively.

The rest of this paper is organized as follows: Section 2 introduces related work about biomedical named entity recognition. Section 3 describes the pattern-based named entity recognition method in detail. Section 4 presents the experiment results of our method and section 5 addresses the conclusions.

## 2. Related Work

Various methods have been developed in clinical natural language processing (NLP) systems. In recent years, machine learning approaches have drawn much attention from the named entities recognition research community. Machine learning approaches usually regard a NER task as a sequence labeling problem. They try to explore the best label sequence, most of the time as BIO (Begin, Inside, Outside) format, for a given input sentence. Among them, Hidden Markov Model (HMM), Support Vector Machines (SVM) and Conditional Random Fields (CRF) are the most frequently used methods (Lafferty et al., 2001; Zhou and Su, 2002; Rössler, 2004). McCallum and Li (2003) proposed a CRF model that achieved an F1 score of 0.84 on the CoNLL-2003 dataset. Recent developments on neural networks boost the CRF-based model by around 10%, improving its capability for public service (Huang et al., 2015; Ma and Hovy, 2016; Gridach, 2017). Despite the superior performance, the approaches still sometimes have trouble in incorporating prior domain knowledge.

The medical field is a specific domain with significant domain knowledge that can be exploited systematically to provide more informative support to application systems. Domain knowledge usually falls into two categories: (1) Discourse knowledge driven by a phenomenon that diagnoses usually stick to a fixed writing style and (2) background knowledge which is captured in medical datasets like UMLS (Bodenreider, 2004), MeSH (Lipscomb, 2000). External knowledge becomes indispensable regarding the requirement of linking named entities to certain concepts.

Jindal and Roth (2013) proposed an Integer Linear Programming approach to incorporate soft global constraints among different named entities. He included the constraint penalty in the training process and improved the performance of the 2010 i2b2/VA dataset. The advantage of incorporating discourse knowledge was also reflected in the application of word embedding (Mikolov et al., 2013), the cornerstone of today's natural language processing field. Zhou et al. (2015) indicated the advantages of introducing continuous word representation to a community question answering system. Emphasized by Wang et al. (2015), the incorporation of continuous word representation allowed the neural model to outperform others on the Google Snippets dataset. Moreover, Character-embedding techniques that followed the same idea pushed the research one step further. Chiu and Nichols (2016) improved the F1 score by 7% on the CoNLL-2003 dataset by adding character embedding. Many of the modern NER systems utilized these techniques and surpassed their counterparts notably (Xie et al., 2017; Xu et al., 2017). In addition, the wide application of the technique (Mikolov et al., 2013; Chung et al., 2014; Rajpurkar et al., 2016; Chen et al., 2017) proved the impact and importance of introducing discourse knowledge.

Similar to the role of discourse knowledge playing in discovering concept-related named entity, background knowledge has a critical influence on named entity normalization. A named entity normalization system proposed by Cho et al. (2017) relied heavily on background knowledge. The key of this system is to utilize a disease/plant name dictionary to augment training data in order to obtain a named entity/concept word representation. As a novel named entity normalization method, Dogan and Lu (2012) trained an abbreviation resolution dictionary based on a phenomenon whereby the complete form and abbreviated form would appear together in biomedical text abstraction. By utilizing automatically studied background knowledge, their method outperformed other state-of-the-art models, e.g., METAMAP (Aronson, 2001), significantly.

Domain adaptation is one of the critical problems in natural language processing. One way to solve this issue is to exploit domain-specific knowledge. Daume and Marcu (2006) proposed MegaM based on an idea that any data could be regarded as a combination of domain-specific and generic features. The evaluation result showed the MegaM's superiority over other baseline methods in terms of performance. Also, by applying MegaM, one could reduce the error rate up to 50%. Following this idea, Daumé (2009) later extended the approach to datasets with arbitrary multiple domains other than just two domains. Kim et al. (2016) implemented this idea with the neural network method and demonstrated a significant improvement over Damué's approach.

## 3. Methodology

Based on a standard dataset obtained from the CHIP 2018 open challenge, this medical named entity recognition research targets three sub-tasks: (1) Identification of primary tumor sites, (2) extraction of primary tumor sizes, and (3) recognition of metastatic tumor sites.

**Figure 1:**
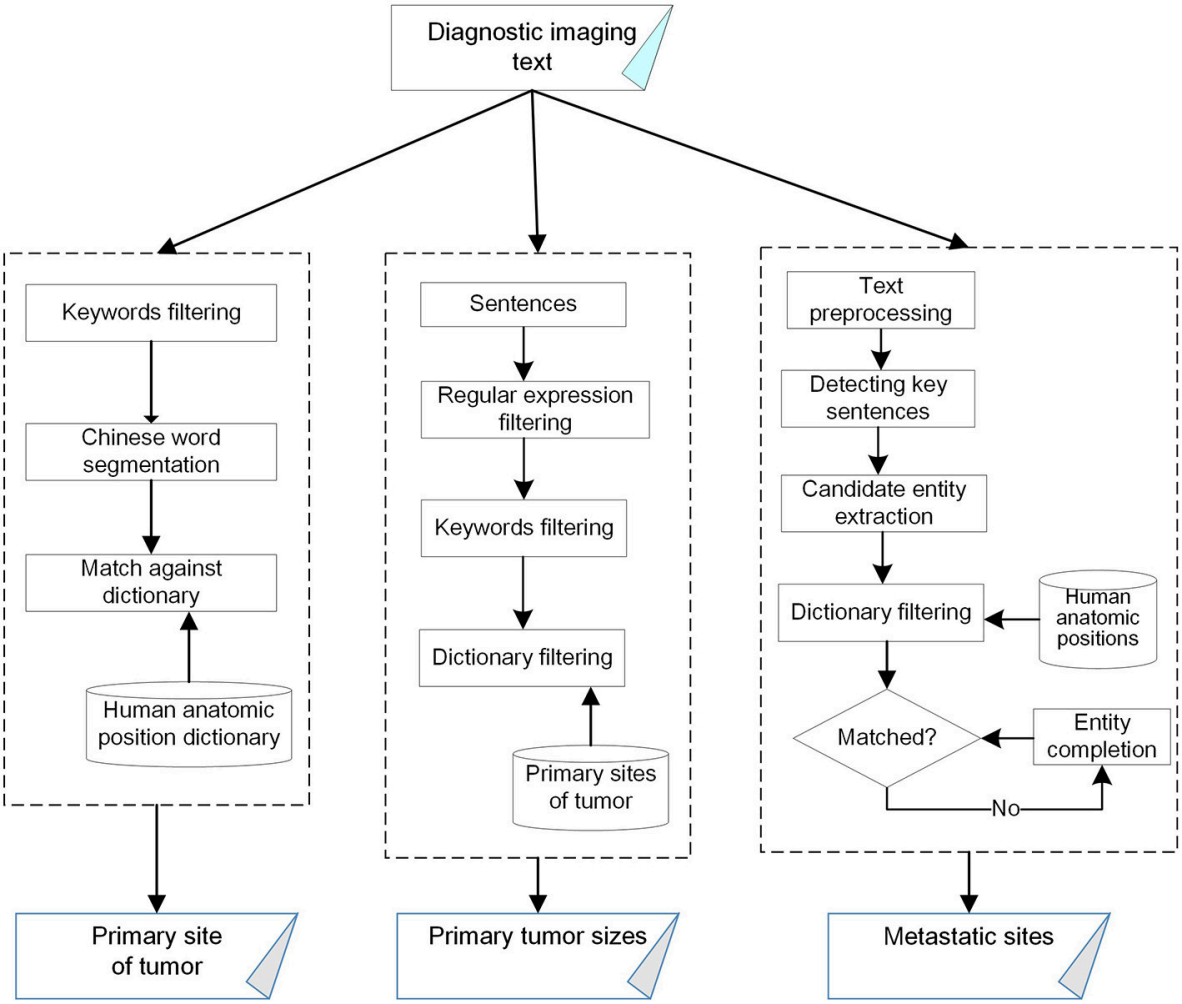
The architecture of our pattern-based biomedical named entity recognition method.

Our method utilizes a pattern-based strategy, which is simple but practical due to the limited volume of data for training complex machine learning models, such as LSTM. The architecture of our method is shown in Figure 1. Our method first applies a Chinese text processing tool named Jieba, which allows users to develop their own word splitting dictionary to produce more accurate word segmentation. Since the generally used version of the tool was trained upon the corpus of People's Daily, which is a one-million-word corpus of Mandarin Chinese from the newspaper People's Daily, we develop a new dictionary containing human anatomic positions to enhance medical word segmentation performance.

### 3.1. Identification of Primary Tumor Sites

According to the task description (Buzhou Tang, 2018b), a primary tumor site is the first anatomic site mentioned in diagnostic imaging text and related to the description of a malignant tumor. For most cases, there is only one primary tumor. Our method thus relies on a common characteristic that cancer entities highly correlate with the appearance of certain indicators which are usually used to describe malignant tumors in Chinese or English, such as “癌” (cancer), “恶性” (malignant), “瘤”(tumor), “MT” (abbreviation of malignant tumor), “CA” (abbreviation of cancer), etc. When an anatomic position and its associated indicating words appear in the same text and this anatomic position is not related to cancer metastasis, the anatomic position is regarded as a primary tumor site. Also, we assume that cancer entities and these indicating words appear in the same sentence context due to their high correlation. Therefore, the method employs a keyword-matching mechanism to filter out irrelevant sentences and reduce computation complexity accordingly. After the filtering process, only sentences which contain target cancer entities are kept. Then, Jieba is applied to split remaining sentences into segments which are subsequently matched against our pre-defined organ dictionary to acquire approximate sites of tumors. In certain cases, a cancer entity may not be sufficient to identify the precise site of a primary cancer. For example, “肺” (lung) can be extracted from “肺癌” (lung cancer). However, it is not the wanted tumor site since lung cancer can refer to any cancer which originated from any part of lung, e.g., superior lobe of left lung. To obtain the specific site, we need to search for a more detailed body part entity from other sentences. We consider the respective body part as where the primary tumor originates from if the detailed entity is a part of the approximate site.

### 3.2. Extraction of Primary Tumor Sizes

This is a method to extract primary tumor size information from diagnostic conclusion text, which usually contains a description of size for each lesion. We design a regular expression-based method for size entity extraction. It is composed of the following steps: (1) Detecting sentences containing size formats, (2) excluding sentences which are not related to primary tumors, and (3) extracting target size mentions with regular expressions.

**Step 1: Detecting sentences containing size formats**. The basis of this method is the strong regularity of size entity expressions. According to the preprocessing of size information in the training dataset, most size entities are represented in particular formats such as “5x4x3CM”, “2.0X2CM”, “5CM*5MM”, “不足 (less than) 5CM”, etc. Therefore, a set of regular expressions is employed to filter out irrelevant sentences. After filtering, sentences containing target expression formats are kept as candidate sentences.

**Step 2: Excluding sentences which are not related to primary tumors**. In most cases, the primary sites of tumor entities obtained from subsection 3.1 and primary tumor size expressions appear in the same sentence. In addition, the size expressions correlate highly with the appearance of certain indicating words, e.g., “高密度影” (high-density shadow), “低密度影” (low-density shadow), “不规则团块” (irregular conglomeration), etc. Therefore, similar to the above entity extraction, the method firstly adopts a keyword-matching mechanism to filter out irrelevant candidate sentences obtained in Step 1. Some tumor site expressions share the same meaning in some cases, such as “左肺门” (left hilum of lung) and “左侧肺门” (left hilum of lung), as well as “右乳腺” (right breast) and “右侧乳腺” (right breast). Therefore, if the primary tumor site obtained from step 1 is about “左肺门” (left hilum of lung), “左侧肺门” (left hilum of lung) should be regarded as a primary site. This method solves the problem of term normalization by expanding some specific primary site entities.

**Step 3: Extracting target size mentions with regular expressions**. After Step 1 and Step 2, we can extract primary tumor size expressions from the remaining sentences by directly using regular expressions. For example, this is a sentence “左肺上叶示一不规则软组织密度灶,大小约1.3CM × 1.7CM,边缘分叶,可见强化。” from a piece of text in the training dataset. The sentence contains a size mention, which can be extracted through a pre-defined regular expression “d+(.d+){0,1}[CcDdMm]{0,1}[Mm]{0,1}[*×*X]d+(.d+){0,1}[CcDdMm]{0,1}[Mm]{0,1}*.” Thus, the target size expression “1.3CMx1.7CM” can be extracted by matching the regular expression to the text.

### 3.3. Recognition of Metastatic Sites of Tumors

The purpose of this sub-task is the extraction of metastatic sites of cancer from diagnostic conclusion text. Metastatic sites of tumors are human anatomic positions highly correlative with keywords that indicate the existence of metastatic cancer, such as “转移” (metastasis), “考虑转移” (considering as metastasis) and “多发转移” (multiple metastasis). Body part descriptions related to such keywords in the same context are considered the metastatic sites of tumors. Based on a set of anatomic positions collected from “Chinese Terms of Human Anatomy” (Human Anatomy and Histology Terminology Committee, 2013) and “Color Atlas of Human Anatomy' (Xingheng Liu, 2007), the identification of metastatic sites of tumors consists of three steps: text preprocessing, key sentence acquisition and missing entities completion.

**Step 1: Text preprocessing**. In diagnostic conclusion text, human anatomic positions are difficult to identify since most of them are presented in a way of describing symptoms rather than stating their locations. For example, “纵隔内淋巴结” (mediastinal lymph node) is usually be presented as “纵隔内多发肿大淋巴结” (mediastinal multiple enlarged lymph node) or “纵隔内小淋巴结” (mediastinal small lymph node). Therefore, our method removes this type of descriptive wording from primitive metastatic entity expressions. In other words, the descriptive words “多发肿大” (multiple enlarged) are removed from the phrase “纵隔内多发肿大淋巴结”.

**Step 2: Key sentence acquisition**. Considering the meaning of metastatic sites of tumor, the metastatic site entities and indicating keywords like “转移” (metastasis) and “多发转移” (multiple metastasis) frequently exist in the same sentence. Hence, the method selects relevant sentences based on this co-occurrence phenomenon.

**Step 3: Missing entity completion**. In many cases, the suffix of multiple entities is mentioned only once if they share the same suffix. For example, both “肺门及前纵隔淋巴结” (pulmonary lymph node and anterior mediastinal lymph node) and “肺门、前纵隔淋巴结” (pulmonary lymph node, anterior mediastinal lymph node) indicate pulmonary lymph node and anterior mediastinal lymph node. Hence, it is incorrect to simply split entities based on conjunctive symbols. Due to this phenomena, a set of rules is applied to identify metastatic entities when conjunctive symbols like “、” (and), “及” (and) or “与” (and) appear in key sentences. For example, “肺门及前纵隔淋巴结” is completed as two entities “肺门淋巴结” (pulmonary lymph node) and “前纵隔淋巴结” (anterior mediastinal lymph node).

## 4. Result and discussion

### 4.1. Datasets

The standard experiment dataset from the CHIP 2018 challenge consists of two sub-datasets: a training dataset and a testing dataset. The training dataset contains 600 pieces of diagnosis text. For each text, the primary tumor sites, primary tumor sizes and metastatic sites are labeled by human annotators from data providers. The 600 pieces of diagnosis text contain 8,401 sentences (14.00 sentences per text on average) in total. A total of 614 primary sites of tumor entities (1.02 entities per text on average), 360 primary tumor size entities (0.60 entities per text on average) and 1,478 metastatic sites of cancer entities (2.46 entities per text on average) are annotated. In addition, for primary site of tumor, there are 351 entities (57.17%) about “肺” (lung) and 225 entities (36.64%) about “乳” (breast). There are 236 different entities (15.97%) among metastatic sites of training dataset and 1,242 entities (84.03%) that appear more than once.

For the testing dataset, 200 pieces of diagnosis text are provided. These text pieces contain 4,934 sentences (24.67 sentences per text on average) in total. A total of 221 primary sites of tumor entities (1.11 entities per text on average), 134 primary tumor size entities (0.67 entities per text on average) and 731 metastatic sites of cancer entities (3.66 entities per text on average) are annotated. In addition, for primary site of tumor, there are 122 entities (55.20%) about “肺” (lung) and 76 entities (34.34%) about “乳” (breast). There are 286 different entities (39.12%) among metastatic sites of the testing dataset and 445 entities (60.88%) appearing more than once. A summary of the training and testing datasets is reported in Table 1.

**Table 1:** The data summary of the training and testing datasets.

Training dataset	Language	#words	#sentences	#primary sites	#pri.tumor sizes
	Chinese	210,073	8,401	614	360
	#texts	#ave.words/text	#ave.sen./text	#ave.pri.sites/text	#ave.pri.tumor sizes/text
	600	350.12	14.00	1.02	0.60
	#metastatic sites	#pri.sites about lung	#pri.sites about breast	#unique meta.sites	#overlapping meta.sites
	1,478	351	225	236	1,242
	#ave.meta.sites/text	%lung/all pri.sites	%breast/all pri.sites	%uni.meta.sites/all meta.sites	%over.meta.sites/all meta.sites
	2.46	57.17%	36.64%	15.97%	84.03%
Testing dataset	Language	#words	#sentences	#primary sites	#pri.tumor sizes
	Chinese	129,100	4,934	221	134
	#texts	#ave.words/text	#ave.sen./text	#ave.pri.sites/text	#ave.pri.tumor sizes/text
	200	645.50	24.67	1.11	0.67
	#metastatic sites	#pri.sites about lung	#pri.sites about breast	#unique meta.sites	#overlapping meta.sites
	731	122	76	286	445
	#ave.meta.sites/text	%lung/all pri.sites	%breast/all pri.sites	%uni.meta.sites/ all meta.sites	%over.meta.sites/ all meta.sites
	3.66	55.20%	34.34%	39.12%	60.88%

### 4.2. Evaluation Metrics

To evaluate our proposed method, we applied three widely used metrics: precision, recall and F1 score. Since this is a typical information extraction task, there are four possible classifications of performance: the information that needs to be extracted is correctly extracted (true positive, TP); the information that does not need to be extracted is wrongly extracted (false positive, FP); the information that needs to be extracted is not extracted (false negative, FN); and the information that does not need to be extracted is not extracted (true negative, TN). Based on the above four classification cases, the meanings of precision, recall and F1 score and their calculation formulas are defined as follows:

(1) $$\begin{array}{rcl} Precision & = & \frac{TP}{TP+FP} \end{array}$$

(2) $$\begin{array}{rcl} Recall & = & \frac{TP}{TP+FN} \end{array}$$

(3) $$\begin{array}{rcl} F1 & = & \frac{2\times Precision\times Recall}{Precision+Recall} \end{array}$$

In this task, we calculate the values of the three evaluation metrics, precision, recall and F1 score, for the three extraction subtasks separately. Meanwhile, we calculate the overall precision, recall and F1 score for the three subtasks as a whole. F1 score considers both the precision rate and the recall rate to compute the score, and the combination of the two can more fully reflect the effectiveness of the method. Therefore, the F1 score is used as the major evaluation criterion. In the calculation of the overall evaluation metrics, the weight of primary location is 0.2, the weight of lesion size is 0.3, and the weight of metastatic site is 0.5 in accordance with the description of this task.

### 4.3. Results

The results of our method on the testing dataset are presented in Table 2. Our method achieves an F1 score greater than 0.7 in all the three subtasks. We note particularly that the F1 score on primary tumor size extraction reaches 0.82. Among them, the performance of primary tumor size extraction is the best and the performance for metastatic site extraction is the worst. The overall unweighted F1 score and the overall weighted F1 score both exceed 0.75.

**Table 2:** Results on the testing dataset.

**Entity identification tasks**	**Precision**	**Recall**	**F1**
primary sites of tumor	0.8250	0.7466	0.7838
primary tumor sizes	0.8301	0.8143	0.8221
metastatic sites	0.8241	0.6712	0.7399
overall	0.8255	0.7113	0.7642
overall weighted	0.8251	0.6973	0.7558

The performances of primary site and metastatic site extractions are not as good as that of primary tumor size extraction on the testing dataset. The major reason may be that human anatomic positions from the training dataset are quite different from the testing dataset. Meanwhile, the human anatomic position dictionary does not have enough coverage. The performance of tumor size extraction is affected slightly with data coverage. Overall, the final performance of this method remains comparatively high (Top 5) among all 92 participants in this challenge.

To verify the robustness, our method runs on different amounts of test data by randomly sampling from the testing dataset. Ten rounds of testing containing a number of test data increasing from 20 to 200, with a step size of 20, are conducted. In each round, it runs 50 times and the average performance is calculated and recorded. Finally, the average overall weighted precision, recall and F1 score of each round are used as evaluation criteria for testing the robustness of this method.

**Figure 2:**
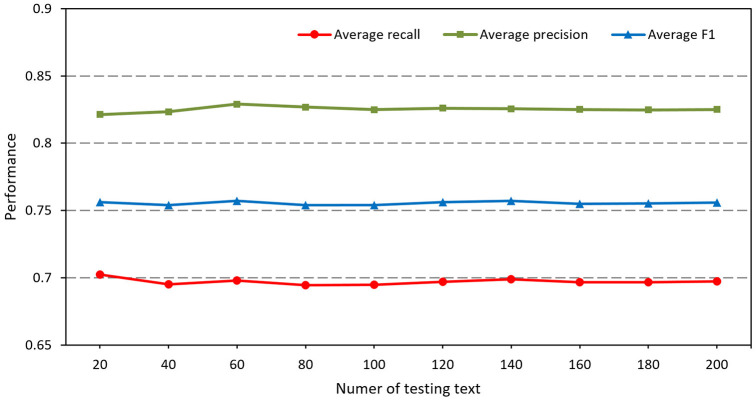
The performance of our method using different numbers of testing text. The average recall, precision and F1 are calculated as the average values for 50 times testing.

As shown in Figure 2, the average overall weighted precision, recall and F1 scores of this method fluctuate slightly when the amounts of test data are small. With an increase in the amount of test data, the performance of the method tends to be more stable. It can be seen that this method has robustness in processing relatively small numbers of data to larger numbers of data.

### 4.4. Error Analysis

There are error cases caused from the results. To understand the hidden reasons, we analyze all the error cases and summarize them according to their respective subtasks.

For the primary sites of tumor identification task, our method fails to identify some primary sites of tumor, which may be caused by the following conditions:

1. When more than one primary tumor site exist in the same phrase context, this method extracts one of primary tumor sites and misses the other. For example, “右乳炎性乳癌术后⋯⋯ ; 右肺中叶磨玻璃结节伴轻度糖代谢增高,考虑为原发性MT可能” (the postoperative treatment of inflammatory breast cancer of the right breast…; in the middle lobe of right lung, ground-glass nodules was accompanied by an increase in mild glucose metabolism, considered as primary MT). The method extracts “右乳” (right breast) which is the first primary site of the tumor from this text and fails to extract the second primary site of the tumor “右肺中叶” (middle lobe of right lung).
2. Long names of human anatomic positions may lead to incomplete extractions of primary tumor sites. In the example “胸下段食管管壁增厚,管腔狭窄,代谢异常活跃,符合食管癌表现” (the tubal wall of the lower thoracic esophagus was thickened, the lumen was narrowed, and the metabolism was abnormally active, which accorded with the appearance of esophageal carcinoma), the primary site of tumor should be “胸下段食管” (lower thoracic esophagus). Nevertheless, our method extracts partial information “食管” (esophagus) as the primary site.
3. Unknown names of human anatomic positions may lead to incorrect extraction. Certain human anatomic positions, such as “胃体部” (gastric body) and “胰尾” (tail of pancreas), do not appear in the training dataset but appear in the testing dataset. Consequently, our method fails to recognize these unknown names without extending our human anatomic position dictionary.

According to the description of the primary tumor sizes extraction task, the extraction of primary tumor sizes relies on the results of the primary tumor site extraction. Therefore, the errors of primary tumor size extraction are mainly caused by the incorrect identification of primary sites of tumors from previous task. In more detail, extraction errors can be divided into the following cases:

(1) Primary tumor size expressions are incorrectly extracted due to the wrong extraction of tumor site, and there is no size information of the wrong primary tumor sites in the text. (2) The extracted primary tumor size expressions are inconsistent with the facts, caused by wrong identifications of primary tumor sites. (3) The numbers of extraction results of primary tumor sizes are insufficient. A diagnostic imaging text may contain more than one primary tumor site, and all of them should be extracted. However, in certain situations, the method extracts only one primary site and misses the size information of another tumor.

For the recognition of metastatic sites, in addition to the incomplete extraction of human anatomic positions, there are several other error cases:

1. Compound words consisting of multiple positions of the human body, where no indicating words such as “及” (and) and “与” (and) are included make it difficult to extract metastatic sites of cancer. For these compounds, their component positions should be split apart during extraction process. For example, “右肺门纵隔多发肿大淋巴结” (right hilar mediastinal multiple enlarged lymph node), two metastatic sites of cancer should be extracted from this phrase: “右肺门淋巴结” (right hilar lymph node) and “纵隔淋巴结” (mediastinal lymph node). However, our method incorrectly extracts them as “右肺门纵隔淋巴结” (right hilar mediastinal lymph node) in the form of compound words.
2. For the sentences with double negative expressions, metastatic sites of cancer are incorrectly extracted. For example, the double negative meaning of “转移不除外” (not excluding metastasis) in the text “左肺多发可疑小结节,转移不除外” (multiple suspicious nodules in the left lung, not excluding metastasis) indicates a possible metastasis. Thus, the metastatic site “左肺” (left lung), which appears before “转移不除外” (not excluding metastasis), should be extracted by this method.
3. Certain human anatomic positions are represented in abbreviations. There is a list of abbreviations for human anatomic positions existing in the text. Without an abbreviation dictionary or the mappings of abbreviations, the method is hard to recognize the abbreviations and normalize them to formal concepts correctly. For example, the abbreviation for the fourth thoracic vertebra is “T4胸椎” (T4 thoracic vertebra), while our method fails to recognize it as a whole entity.

## 5. Conclusions

In this paper, we proposed a pattern-based method to extract primary tumor sites, the size of primary tumors and metastatic sites from diagnostic imaging test. Based on a standard dataset provided by the CHIP 2018 open challenge, the method was tested and evaluated. The results demonstrate that the method achieves a relatively high performance, with an overall weighted F1 score of 0.7558 on the testing dataset. The error cases were fully analyzed, and further improvement strategies are under design. This method contributes to extracting certain entities and expressions from unstructured Chinese electronic medical record text.

## Ethics Statement

The study is based on the publicly available datasets and an ethics approval was not required for the study as per applicable institutional and national guidelines and regulations.

## Author Contributions

ZL, JC, and ZX led the method design and experiment implementation. ZL and ZX performed the statistical analysis. ZL, JC, ZX, and YC wrote sections of the manuscript. TH provided theoretical guidance, result review, and paper revision. All authors read and approved the final manuscript.

### Conflict of Interest Statement

The authors declare that the research was conducted in the absence of any commercial or financial relationships that could be construed as a potential conflict of interest.
